# Complete chloroplast genome sequence and annotation of *Actinodaphne lecomtei* C.K.Allen, 1938 (Lauraceae)

**DOI:** 10.1080/23802359.2022.2054382

**Published:** 2022-03-28

**Authors:** Zimeng Chen, Li He, Song Weicai, Wenbo Shi, Qin Gong, Chao Shi

**Affiliations:** a College of Marine Science and Biological Engineering, Qingdao University of Science and Technology, Qingdao, China; b College of Art, Qingdao University of Science and Technology, Qingdao, China; c Plant Germplasm and Genomics Center, Germplasm Bank of Wild Species in Southwest China, Kunming Institute of Botany, The Chinese Academy of Sciences, Kunming, China

**Keywords:** *Actinodaphne lecomtei*, Lauraceae, chloroplast genome, phylogenetic relationships

## Abstract

*Actinodaphne lecomtei* C.K.Allen, 1938 is an evergreen tree of the Lauraceae family and grows at the mountainous areas of southwestern China. In this study, we presented the first complete chloroplast genome sequence of *A. lecomtei.* We analyzed the chloroplast genome structure of *A. lecomtei* and performed a phylogenetic analysis. The complete chloroplast genome of *A. lecomtei* was 152,863 bp in length which contains a large single-copy (LSC) region of 93,763 bp, a small single-copy (SSC) region of 18,814 bp, and two inverted repeat (IR) regions of 20,143 bp. The analysis identified 128 genes, comprised of 84 protein-coding genes, 36 tRNAs, and eight rRNAs. The GC content of *A. lecomtei* complete chloroplast genome was 39.1%. The phylogenetic analysis result demonstrated that *A. lecomtei* was closely related to *A. obovate.*

*Actinodaphne lecomtei* C.K.Allen, 1938 is an evergreen tree of the Lauraceae family. It is mainly found in the provinces of Sichuan, Guizhou, and Yunnan in China and grows in mountains at 650*–*1800 m above sea level. Among the applications of *A. lecomtei* are making of furniture from its wood and lubricating of machines using its oil (Blanchard 2008; Ou et al. 2014). Owing to its highly conserved structure and low mutation rate, chloroplast genome has been extensively used to understand evolution and gene structure (Ebrahimi et al. 2021). To date, however, the chloroplast genome of *A. lecomtei* has not been determined. In this study, we presented the first complete chloroplast genome sequence of *A. lecomtei.* We analyzed the chloroplast genome structure of *A. lecomtei* and performed a phylogenetic analysis. This study will be useful for future studies and phylogenetic analyses of chloroplast genomes in Lauraceae species (Song et al. 2018).

The fresh samples of *A. lecomtei* were collected from Yunnan Province, China (24°23′N, 102°10′E). The study was conducted with consent from the local government and the Kunming Institute of Botany, Chinese Academy of Sciences. The voucher specimen was deposited in Qingdao University of Science and Technology (Chao Shi, chsh1111@aliyun.com) under the specimen code AL202119. Approximately, 30 g of fresh mature leaves of *A. lecomtei* were used to extract chloroplast DNA using the modified high salt method previously reported (Shi et al. 2012). Both the quantity and quality of the extracted DNA was assessed spectrophotometrically and the integrity was assessed using 1% (w/v) agarose gel electrophoresis. DNA of high quality was sent to Novogene (Beijing, China) for genomic library construction and sequencing using the Illumina HiSeq platform (Illumina, San Diego, CA). About 4.8 Gb high quality, 2 × 150 bp pair-end raw reads were obtained and were used to assemble the complete chloroplast genome of *A. lecomtei* (Wang et al. 2018). The chloroplast genome of *A. lecomtei* was de novo assembled through NOVOPlasty v4.3.1 (Dierckxsens et al. 2017) and was annotated by GeSeq (Tillich et al. 2017). Sequin was used to manually correct codons and gene boundaries.

The chloroplast genome of *A. lecomtei* (GenBank accession MZ442604) presented a typical quadripartite structure (Wicke et al. 2011) with a total length of 152,863 bp which contains a large single-copy (LSC) region of 93,763 bp, a small single-copy (SSC) region of 18,814 bp, and two inverted repeat (IR) regions of 20,143 bp. The analysis identified 128 genes, comprised of 84 protein-coding genes, 36 tRNAs, and eight rRNAs. The complete chloroplast genome of *A. lecomtei* had the GC content of 39.1%. To reveal the evolutionary relationships between *A. lecomtei* and other Lauraceae, *Sinopora hongkongensis* was used as outgroups, together with 13 Lauraceae species to construct a phylogenetic tree (Nie et al. 2007; Zhang et al. 2021). To create sequence alignments for the construction of phylogenetic trees, MAFFT v725 (Katoh and Standley 2013) was used. Then the GTR-GAMMA (GTR + G) model was selected by applying the Bayesian information criterion (BIC) by Modeltest (Posada and Crandall 1998). Finally, MEGA-X software (Kumar et al. 2018) was used to perform 1000 bootstrap replications using the maximum-likelihood (ML) method. Phylogenetic analysis showed that *A. lecomtei* is closely related to *A. obovate* (Figure 1). This result was similar to previous studies (Fijridiyanto and Murakami 2009; Song et al. 2017).

**Figure 1.**
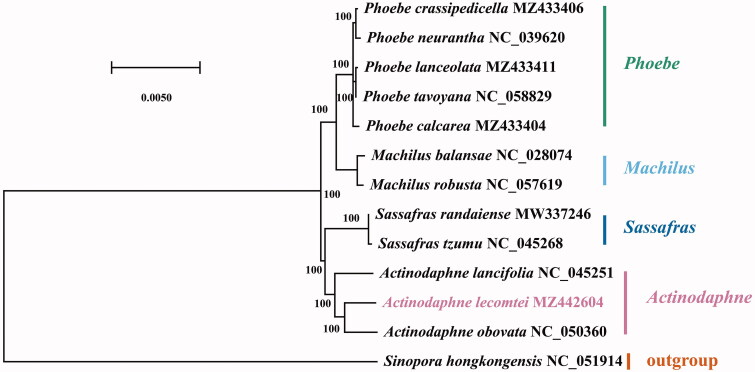
The maximum-likelihood tree states the phylogenetic position in Lauraceae of *Actinodaphne lecomtei*, with the number on each node denoting the bootstrap support value. The species is followed by the chloroplast genome accession number that was used by GenBank.

## Data Availability

The genome sequence data that support the findings of this study are openly available in GenBank of NCBI at https://www.ncbi.nlm.nih.gov/ under the accession number MZ442604. The associated BioProject, SRA, and Bio-Sample numbers are PRJNA786749, SRR17153750, and SAMN23720001, respectively.
